# Prokaryotic homeostasis – a solution to thrive and survive

**DOI:** 10.3389/fmolb.2025.1704789

**Published:** 2025-10-24

**Authors:** Sylwia Barańska, Lidia Boss, Filip Gąsior, Monika Glinkowska, Barbara Kędzierska, Monika Maciąg-Dorszyńska, Dariusz Nowicki, Katarzyna Potrykus, Agnieszka Szalewska-Pałasz

**Affiliations:** Department of Bacterial Molecular Genetics, Faculty of Biology, University of Gdańsk, Gdańsk, Poland

**Keywords:** homeostasis, DNA structure and repair, metabolism, toxin-antitoxin systems, bacteriophage, stress response, second messengers, biofilm

## Abstract

Bacteria have been generally greatly overlooked in the aspect of intra- and extra-cellular homeostasis, and yet, since they have evolved intricate processes and mechanisms allowing them not only to stay alive but also thrive in favorable and unfavorable environments alike, they should be considered as a close-to-ideal example of single-cell homeostasis. The bacterial responses aimed at maintaining homeostasis, while adjusting and reacting smoothly and swiftly to any changes inside and outside the cell, involve complex transcriptional networks regulated by second messengers and DNA topology, but also influenced by the presence of prophages and toxin-antitoxin systems. Their adjustment to nutrient availability also involves homeostasis in energy-related processes, such as central carbon metabolism, and crucial ion acquisition, e.g., iron. The genome stability, which is indispensable to maintain a given organisms’ functions, is achieved by control of DNA replication and repair. Furthermore, bacteria can form multicellular structures (biofilms), where homeostasis is achieved at several different levels and provides bacteria with higher chances of survival and colonization of new niches and locations. Precise correlation between the above-mentioned cellular processes makes bacteria highly intriguing objects of studies. Homeostasis is the most important basis of their life-style flexibility, thus understanding of these processes is indispensable for both: the basic and applied sciences. For example, understanding how chromosomal architecture and DNA topology coordinate global gene expression is essential for optimizing strain engineering and synthetic biology applications. Moreover, bacterial homeostasis regulatory processes can be employed as targets for antibacterial agents and prospective therapies.

## Graphical Abstract

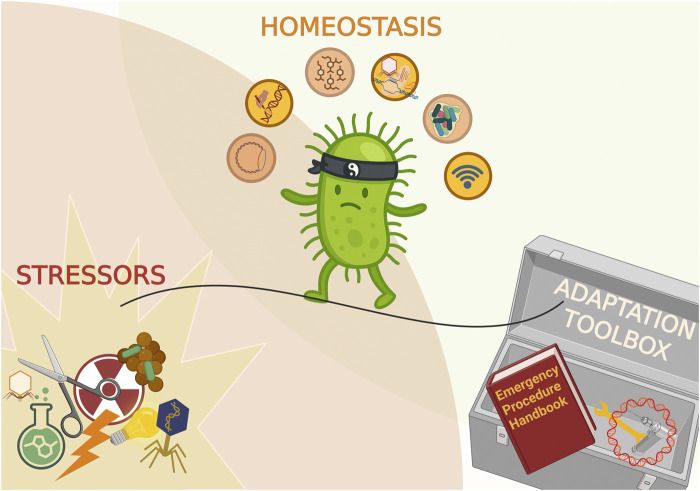

## Introduction

Bacteria are rarely presented in the context of cellular homeostasis. However, as single-cell organisms they have mastered the asset of maintaining intracellular balance and instant adaptations to the environment and numerous stresses. Such adjustments are indispensable for prokaryotic organisms to increase their chances of survival. Thus, many complex mechanisms have evolved in bacteria to overcome adverse conditions and to optimally use available resources. These processes are integrated in the cell as a response to extracellular signals which provide information about potential stresses, nutrient limitations or indicate the need to readjust cellular processes in response to major alternations in the cell’s lifestyle.

Bacteria can live in a variety of environments, including those that are very poor and limited in nutrients. To meet the need for energy saving, regulation of homeostasis preferentially occurs at the level of gene expression. Numerous regulators precisely control the choice of relevant genes and operons available for the transcription process. In this regulation, not only canonical protein regulators are involved. Bacteria utilize a variety of low-molecular-weight non-proteinaceous molecules, called alarmones, which serve as second messengers for coordination of gene expression patterns to ensure relevant flexibility of cellular responses. Also, the organization of bacterial genetic material (bacterial chromosome and extrachromosomal elements) serves as a route to grant access to the transcriptional machinery of specific DNA regions in response to the environmental cues. Moreover, bacteria are susceptible to DNA damage as the result of environmental factors, including antibacterial compounds, thus DNA repair processes are a major part of genomic integrity. The complex network to integrate environmental signals also involves carbon metabolism to optimize fundamental processes of macromolecule synthesis and overall energy balance. The rate of bacterial growth has to be correlated with DNA replication to maintain genetic information stability. Also, to achieve optimal growth, these single cell organisms need to balance the uptake of crucial compounds to maintain their stable concentrations in the cell. A well-known example of such a process is iron homeostasis which involves multi-step control of iron acquisition, storage and usage; moreover, it plays a very important role in bacterial pathogenesis. In addition, the presence of specific genetic elements, such as toxin-antitoxin systems or prophages is not only a burden for a cell that is inherited from previous generations but apparently it plays an important role in diverse cellular processes. Also, in addition to the single-cell planktonic state, bacteria form multicellular communities, i.e., biofilms, where interactions among cells affect cellular processes.

**FIGURE 1:**
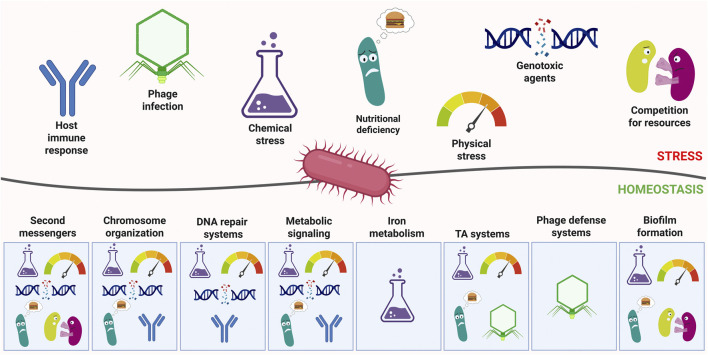
General overview of stresses faced by bacteria and the relevant countermeasures. Major stress categories are depicted as pictograms above the line representing the interaction between bacteria and the environment. Below, the systems controlling homeostasis are depicted as responsive to relevant stresses. Created in BioRender. Potrykus (2025) https://BioRender.com/lsab5zb.

This review presents recent advances and developments in the knowledge of specific processes necessary for cellular homeostasis of prokaryotic organisms (presented in Figure 1). Integration of various cellular processes, and the flow between them, especially in response to stress, is key not only for bacterial cells to survive under adverse circumstances but also to thrive under permissive conditions, taking advantages of available options.

## Second messengers: important molecules for maintaining homeostasis acting by relaying information about environment to bacterial cells

Second messengers play an important role in relaying information about the environmental status to the bacterial cell. In general, a specific signal triggers production of a modified nucleotide or molecule that in turn influences cellular metabolism or gene expression. Examples of nucleotide second messengers in bacteria include cyclic (e.g., cAMP, cGMP, c-di-GMP, and c-di-AMP) and non-cyclic derivatives (e.g., (p)ppGpp, (p)ppApp, Ap_4_A, and ZTP). Each of them corresponds to distinct conditions and is synthesized and hydrolyzed by a specific set of enzymes, since it is important not only to synthesize them quickly but also to swiftly degrade them once conditions change so that the cell can rewire its gene expression and metabolism accordingly.

The first to be discovered was cAMP (3′5′cyclic adenosine monophosphate) that in *Escherichia coli* and other Gram(−) bacteria governs carbon source utilization through binding to a pleiotropic transcriptional regulator CRP. This nucleotide derivative is synthesized by adenylate cyclase (Cya) and is degraded by a specific phosphodiesterase (CpdA). In *E. coli*, cAMP-CRP regulates expression of over 100 genes allowing for flexibility in different nutritional source utilization, as well as for promoting growth for as long as possible (reviewed in Hengge (2020)). Its structural analog, cGMP (3′5′cyclic guanosine monophosphate), is much less studied in bacteria, however it too was shown to act as a second messenger in α-proteobacteria and cyanobacteria where it was reported to participate in dormant cell formation and UV stress adaptation, respectively (reviewed in Gomelsky and Galperin (2013)).

On the other hand, the well-known ppGpp and pppGpp alarmones (guanosine 5‵diphosphate-3‵diphosphate and guanosine 5‵triphosphate-3‵diphosphate, respectively, collectively referred to as (p)ppGpp), are the effectors of the so-called stringent response whose purpose is to limit cellular growth and promote survival strategies, including virulence genes’ expression in case of pathogenic bacteria (reviewed in e.g., Potrykus and Cashel (2008), Dalebroux et al. (2010), Irving et al. (2021)). This response is activated upon amino acid-, carbon-, nitrogen-, phosphate-, lipid-, and iron-limitation, as well as under oxidative and acid stress (e.g., Potrykus and Cashel, 2008; Irving et al., 2021). The (p)ppGpp metabolism is generally carried out by RelA/SpoT Homolog proteins (RSH) which can be divided into “long” (i.e., possessing both, an enzymatically active synthetase and hydrolase domain, along with a regulatory domain) and “short” enzymes (possessing only the synthetase (SAS–small alarmone synthetase) or hydrolase (SAH–small alarmone hydrolase) domains, with no apparent regulatory domain). Examples of “long” bifunctional RSH proteins are SpoT in *E. coli* and Rel in *Bacillus subtilis*. In addition, most β- and γ-proteobacteria also possess a synthetase-only long RSH (e.g., RelA in *E. coli*) (reviewed in Bange et al. (2021)), while *Acinetobacter baumanii* contains a hydrolase-only long RSH (Tamman et al., 2023). Importantly, (p)ppGpp and/or RSH enzymes have been identified in all bacterial species studied so far.

The (p)ppGpp’s effects on transcription can be direct due to its binding to the RNA polymerase (e.g., in *E. coli*) or indirect due to (p)ppGpp’s interference with purine metabolism causing a drop in cellular GTP levels (e.g., in Firmicutes). Besides affecting gene transcription, (p)ppGpp was also shown to inhibit DNA replication, limit translation, and promote DNA repair (reviewed in Rasouly et al. (2017); Irving et al. (2021)). It should be also mentioned, that the (p)ppGpp family of second messengers also includes pGpp (guanosine 5‵monophosphate-3‵diphosphate) which was thought at first to be a “lesser” version of ppGpp, but recently has been shown to be synthesized under distinct conditions and to have its own distinct targets (Malik et al., 2023). In addition, adenosine derivatives that are structurally related to (p)ppGpp–pppApp and ppApp are recently gaining attention as possible second messengers as well. They were first discovered in *B. subtilis* in the 1970’s, but for many years were viewed as artefacts. Yet, recently they were demonstrated to be synthesized and degraded by various RSH enzymes (including SAS’s and SAH’s) that were originally thought to only metabolize (p)ppGpp, as well as synthesized by a *Pseudomonas aeruginosa* encoded interbacterial toxin Tas1 and degraded by a specific class of SAH enzymes for which they seem to be the only substrate (Ahmad et al., 2019; Ahmad et al., 2023; Sobala et al., 2019; Fung et al., 2020; Irving et al., 2021). Although ppApp was shown to bind to *E. coli* RNA polymerase and to have an opposite effect on transcription to (p)ppGpp *in vitro*, its physiological role, as well as conditions triggering its synthesis remain to be elucidated (Bruhn-Olszewska et al., 2018; Irving et al., 2021).

Other nucleotide second messengers whose involvement in cellular homeostasis cannot be omitted include c-di-GMP and c-di-AMP. The c-di-GMP (bis-(3′,5′)-cyclic di-guanosine monophosphate) is present in both, Gram(−) and Gram(+) bacteria and is generally known to regulate bacterial lifestyle transition from motile to sedentary (adhesive), biofilm formation, cell cycle and virulence (Pesavento and Hengge, 2009; Opoku-Temeng and Sintim, 2017). Synthesis of this second messenger is carried out by enzymes from the diguanylate cyclase family (DGCs) and there are known two distinct families of c-di-GMP-specific phosphodiesterases (EAL and HD-GYP domain enzymes) (Hengge et al., 2016). It has been reported that c-di-GMP synthesis is triggered by a variety of environmental cues and its targets include enzymes, transcription factors and riboswitches (Opoku-Temeng and Sintim, 2017). Intriguingly, some bacteria can encode many different types of c-di-GMP synthetases and phosphodiesterases, e.g., *P. aeruginosa* harbors over thirty different DGC’s which suggests they may each respond to a different environmental condition (Opoku-Temeng and Sintim, 2017).

On the other hand, c-di-AMP (bis-(3′,5′)-cyclic di-adenosine monophosphate) is responsible for cell wall homeostasis, osmoregulation, DNA integrity and DNA repair, genetic competence, sporulation, and biofilm formation (Stülke and Krüger, 2020; Hengge et al., 2023; Herzberg et al., 2023). The c-di-AMP synthesis is catalyzed by specific diadenylate cyclases (most bacteria encode one such enzyme, CdaA) in response to potassium availability and the choice of nitrogen source, and hydrolysis is facilitated by dedicated phosphodiesterases (Stülke and Krüger, 2020; Herzberg et al., 2023). The diadenylate cyclases were identified in Actinobacteria, *Chlamydia*, δ-proteobacteria, Firmicutes, Spirochaetes, and the Cytophaga/*Flavobacterium*/*Bacteroides* group (Stülke and Krüger, 2020). Interestingly, c-di-AMP can exert its action by binding to riboswitches, proteins or even both, as evidenced for *B. subtilis* KtrAB and KimA potassium transporter proteins that are inhibited at the level of enzymatic activity as well as at the level of their translation (Herzberg et al., 2023). In addition, this nucleotide has been shown to be tightly correlated with cellular metabolism in *B. subtilis* and *Listeria monocytogenes*, where it controls pyruvate carboxylase activity in response to cellular potassium levels (Herzberg et al., 2023).

There are also other nucleotide derivatives recently emerging as second messengers and involved in maintaining cellular homeostasis. For example, Ap_4_A (di-adenosine tetraphosphate) has been reported to act as a second messenger in *B. subtilis* where it inhibits purine biosynthesis (Giammarinaro et al., 2022; Young and Wang, 2024). Another example is ZTP (5-amino-4-imidazole carboxamide riboside 5′triphosphate) which is essential during zinc deficiency in *B. subtilis* as it helps to sustain folate synthesis under such conditions (Chandrangsu et al., 2019).

Finally, it has to be highlighted that there is a well-documented crosstalk between several second messenger systems, such as (p)ppGpp and c-di-AMP; c-di-GMP with cAMP and (p)ppGpp; cAMP and (p)ppGpp; (p)ppGpp and pGpp; (p)ppGpp and Ap4A (for details see the following and references therein: Pesavento and Hengge (2009), Meyer et al. (2021), Ro et al. (2021), Fung et al. (2023), Herzberg et al. (2023)). This indicates that second messenger systems are tightly intertwined with each other and deeply embedded in bacterial regulatory networks, allowing for fine-tuning of bacterial response to different environmental cues and maintaining cell homeostasis.

## Chromosomes: their structure, organization and role in the context of homeostasis

The life cycle of a bacterial cell necessitates genome duplication and segregation prior to cell division. Simultaneously, cell growth requires coordinated expression of numerous genes, predominantly regulated at the level of transcription initiation. Moreover, dynamic reshuffling of transcriptional program, essential for the cell to adapt to environmental changes, implies that DNA topology must be sufficiently flexible to allow rapid reorganization of the transcriptional machinery.

Inside the bacterial cell, genetic material is compacted by approximately three orders of magnitude relative to its linear length. Chromosomal DNA is a long and elastic polymer with a double-helical structure comprising 10.5 base pairs per helical turn in its predominant B-form. Processes involving strand separation, such as transcription and replication, introduce supercoiling (SC), which manifests as either twist (helical overwinding or underwinding) or writhe (three-dimensional coiling of the DNA helix). For instance, RNA polymerase (RNAP) generates positive supercoiling ahead and negative supercoiling behind the transcription complex (so called twin-domain) (Liu and Wang, 1987). Similarly, the advancing replisome introduces strong positive supercoiling ahead of the replication fork (Postow et al., 2001). Hence, keeping the genome homeostasis and preventing inhibition of DNA transactions is by itself challenging. Moreover, supercoiling-transcription relationship is bi-directional, the transcription process itself—but also DNA replication—have been shown to be sensitive to topological constraints. Thus, in the context of cellular homeostasis, bacterial chromosome biology can be viewed through two complementary lenses: (1) the mechanisms that shape chromosome architecture and maintain topological homeostasis; and (2) the role of chromosome structure in regulating gene expression and other DNA-dependent processes in response to environmental conditions.

In most bacterial species, the genome is comprised of single circular DNA molecule organized into two replichores along the (single) origin-to-terminus of replication axis, reflecting bi-directional replication (Touchon and Rocha, 2016; Ponndara et al., 2025). The development of chromosome conformation capture techniques (Hoareau et al., 2024) has significantly advanced our understanding of intracellular chromosome architecture.

These studies highlighted the role of transcription in shaping the 3D architecture of bacterial chromosomes. Chromosomal Interaction Domains (CIDs)—regions with elevated contact frequencies (similar to Topologically Associating Domains - TADs in eukaryotic cells) (Le et al., 2013) were identified in all bacterial genomes studied to date (Ponndara et al., 2025). CID boundaries are frequently associated with long, highly transcribed operons, indicating that chromosome folding is modulated by active transcription.

More recent studies in *E. coli* revealed that the chromosome is organized into domains aligned with transcriptionally active genes. These transcription-induced domains (TIDs) promote contacts between loci separated by several tens of kilobases and are separated by non-transcribed regions (Ponndara et al., 2025). A single highly active transcription unit is sufficient to generate a discrete domain. TID formation was associated with relocation to the nucleoid periphery, indicating that transcription influences both the topology and spatial positioning of transcribed loci (Bignaud et al., 2024). In addition, a new ultra-high-resolution study of *E. coli* chromosomes showed operon-sized chromosomal interaction domains (OPCIDs), which are strictly dependent on transcription (Gavrilov et al., 2025). The relationship between TIDs and OPCIDs remains to be fully elucidated.

Transcription also drives distribution of DNA supercoiling in the *E. coli* genome (Visser et al., 2022). Genome-wide supercoiling analyses showed that highly transcribed rRNA operons generate extensive twin-supercoiled domains (∼25 kb), considerably exceeding the length of similar domains in eukaryotes. This study also revealed that, while bacterial chromosomes are negatively supercoiled on average, the origin-proximal half exhibits more negative supercoiling than the terminus-proximal half. This observation supports earlier models suggesting that negative supercoiling gradients along replichores contribute to global gene expression regulation (Sobetzko et al., 2013; Menzel and Gellert, 2024).

The extended twin-domains formed by highly transcribed genes suggest that transcription-driven supercoiling can overwhelm the buffering capacity of topoisomerases. Replication complex imposes even greater topological stress, generating strong positive supercoiling and leading to intertwining of sister chromatids (catenation). *E. coli* encodes four topoisomerases: Topo I, which relaxes negative supercoiling arising during transcription; DNA gyrase, which removes positive supercoils and introduces negative supercoils in an ATP-dependent manner; and Topo IV and Topo III, which are primarily involved in decatenation. The balance between Topo I and gyrase is primarily responsible for maintaining average superhelicity. Recent studies highlight how topoisomerase activities are spatially and temporally coordinated through protein–protein interactions (Kim and Guo, 2024). For example, Topo I interacts with the β′ subunit of RNA polymerase. Disruption of this interaction leads to excessive negative supercoiling and cell death due to R-loop formation (Sutormin et al., 2022) which stall replication and transcription (Drolet and Brochu, 2019).

Similarly, the timing of decatenation by Topo IV is regulated by interactions with several chromosome-organizing proteins that coordinate chromosome segregation with cell division (Waldminghaus and Skarstad, 2009; Joshi et al., 2013; Nolivos et al., 2016; Fisher et al., 2021; Helgesen et al., 2021; Sutormin et al., 2023; Kim and Guo, 2024).

In addition to topoisomerases, nucleoid-associated proteins (NAPs) play important roles in chromosome organization (Dame et al., 2020; Hustmyer and Landick, 2024). With the exception of the HU family, NAP repertoires vary across bacterial species. NAPs, while binding DNA with variable degree of sequence-specificity, can bend, bridge, loop, or wrap DNA, modulating gene expression and replication. For a long time, NAPs were considered the primary determinants of global chromosome architecture. However, only HU deletion has been shown to significantly disrupt overall chromosomal structure, while other NAPs exert more localized effects (Nolivos and Sherratt, 2014; Lioy et al., 2018). Recent study revealed chromosomal hairpins (CHINs) and hairpin domains (CHIDs) dependent on H-NS (primarily) and its paralog StpA. Consistently with previously demonstrated H-NS role (Rashid and Dame, 2023), these structures map to horizontally transferred genes (HTGs) and are critical for silencing of foreign DNA (Gavrilov et al., 2025). NAPs composition and properties are also modulated by environmental cues which plays an important role in regulating various processes and maintenance of cellular homeostasis (Ge et al., 2025).

Over the past two decades, DNA supercoiling has emerged as a key global regulator of gene expression in bacteria (Dorman and Dorman, 2016; Martis et al., 2019; Le Berre et al., 2022). Changes of DNA superhelicity participate in orchestrating response of bacterial cells to many stress conditions, including those that pathogenic bacteria come across in host organisms (Dorman et al., 2016; Martis et al., 2019).

The underlying mechanisms include altered expression and activity of NAPs and topoisomerases (Menzel and Gellert, 2024; Hustmyer and Landick, 2024; Ge et al., 2025) modulation of topoisomerase activity (Zhou et al., 2018), and changes in the [ATP]/[ADP] ratio, which directly impacts gyrase function (van Workum et al., 1996). For example, osmotic stress increases [ATP]/[ADP], enhancing gyrase activity and negative supercoiling (Hsieh et al., 1991), while anaerobic conditions decrease it (Weinstein-Fischer et al., 2000). These shifts in supercoiling can influence both transcription initiation and elongation. The most obvious effect of DNA topology relies on facilitating strands unwinding and hence, open complex formation by negative superhelicity (Martis et al., 2019).

Thus, the bacterial chromosome conveys not only genetic information, but also structural information that responds to environmental signals and modulates transcriptional outputs (Muskhelishvili et al., 2024). Several studies using topoisomerase inhibitors identified large sets of genes responsive to altered supercoiling (Peter et al., 2004; Blot et al., 2006; Xuejiao et al., 2015; Pineau et al., 2022). However, the determinants of supercoiling sensitivity remain incompletely understood.

Some research links promoter architecture to this phenomenon, showing that sequence and length of certain bacterial promoter elements (discriminator and spacer) play critical roles in modulating promoter responsiveness to superhelical stress (Klein et al., 2021; Raphaël et al., 2021; Forquet et al., 2022). Other studies emphasize the importance of local genomic context for supercoiling-driven transcription regulation, also in eukaryotic cells (Kouzine et al., 2013; Sobetzko, 2016; El Houdaigui et al., 2019; Martis et al., 2019). Given the bidirectional influence between transcription and supercoiling, adjacent promoters can affect each other in an orientation-dependent manner: divergent promoters may mutually activate, while convergent promoters often repress one another. Synthetic promoter constructs confirmed this model, revealing strong temporal coupling between tandem promoter pairs (Moulin et al., 2005; Sobetzko, 2016). Biophysical models further support the idea that global changes in DNA topology, under control of cellular physiology (i.e., [ATP]/[ADP] ratio), enable finely tuned regulation based on local promoter interactions (El Houdaigui et al., 2019).

In the longest-running bacterial evolution experiment, *E. coli* strains selected for enhanced fitness displayed increased negative supercoiling (El Houdaigui et al., 2019). Expression profiles of the evolved strains in comparison to their ancestor suggest that changes in transcription are related to local gene context and orientation, since the mutants showed stronger activation of divergent genes. This result underscores the role of topological coupling in shaping chromosomal gene arrangement over evolutionary timescales (El Houdaigui et al., 2019).

In summary, bacteria exploit the biophysical properties of DNA and an evolutionarily optimized genome organization to coordinate gene expression. An emerging aspect of this regulatory framework is the influence of gene distance from the origin of replication on gene activity, demonstrated both in population studies (Slager and Veening, 2016; Yubero and Poyatos, 2020; Teufel et al., 2023) and single-cell transcriptome analysis (Wang et al., 2019; Pountain et al., 2024). During the cell cycle gene copy number in the cell doubles as the chromosomal DNA is replicated. Consequently, genes closer to the origin are present in more copies for a longer part of the cell cycle than genes close to the terminus, particularly in fast-growing bacteria with multiple ongoing replication rounds. This results in significantly higher expression levels for the origin-proximal genes. Relocating the origin of replication to the terminus demonstrated that copy number alone can account for expression patterns of most genes during exponential growth (Teufel et al., 2023). Analyses of thousands of transcriptomic datasets confirmed that gene position along the Ori–Ter axis is a major predictor of expression levels, especially under rapid growth (Kosmidis et al., 2020). Hence, the distance from the replication origin can be a strong factor driving evolution of chromosomal gene localization. Indeed, conservation of gene order along the Ori-Ter axis was demonstrated before (Sobetzko et al., 2012). Furthermore, recent studies across hundreds of bacterial genomes revealed that over half of gene families show conserved positional biases, especially in fast growing species (Hu et al., 2025).

## The guardians of the genome: DNA repair mechanisms in bacterial homeostasis and pathogenesis

Bacteria constantly face the challenge of maintaining genomic stability in environments filled with genotoxic agents. These include ultraviolet (UV) radiation, reactive oxygen species (ROS), reactive nitrogen species (RNS), hydrogen peroxide (H_2_O_2_), and other toxic metabolic products (Silva-Júnior et al., 2011; Santos et al., 2012; Fasnacht and Polacek, 2021). Such agents can cause a wide array of DNA lesions, including base modifications (e.g., methylation, deamination), depurination, single-strand breaks (SSBs), and the more severe double-strand breaks (DSBs), all of which threaten cellular survival (Gupta et al., 2011; Brzostek et al., 2014).

To combat these threats and maintain genomic homeostasis, bacteria have evolved a diverse set of highly conserved DNA repair mechanisms - these include base excision repair (BER), nucleotide excision repair (NER), mismatch repair (MMR), homologous recombination (HR), non-homologous end joining (NHEJ), single-strand annealing (SSA), and the global SOS response system (Fukui, 2010; Morita et al., 2010; Wozniak and Simmons, 2022). These systems play a central role not only in survival under stressful conditions but also in ensuring accurate replication, genome transmission, and, in the case of pathogens, virulence and persistence in hostile host environments.

The importance of these mechanisms is particularly pronounced in pathogenic bacteria, which encounter intense oxidative and nitrosative stress during infection. *Mycobacterium tuberculosis*, the causative agent of tuberculosis, serves as a leading example of a pathogen whose survival within host macrophages depends on the effectiveness of its DNA repair machinery. Macrophages generate large amounts of ROSs and RNSs as part of their antimicrobial defense, subjecting internalized bacteria to oxidative DNA damage (Brzostek et al., 2014). Studies have shown that in such an environment, the DNA of *M. tuberculosis* undergoes extensive base modifications, including methylation, deamination, and depurination, frequently resulting in SSBs and DSBs (Gupta et al., 2011; Brzostek et al., 2014).

The indispensability of efficient DNA repair in *M. tuberculosis* is underscored by genetic studies involving mutants deficient in key components of HR and NHEJ pathways. For instance, *M. tuberculosis* strains lacking the RecA protein (essential for HR) or Ku and LigD proteins (central to NHEJ) fail to survive within human macrophages, but interestingly, the presence of at least one of these two pathways is sufficient to restore bacterial viability, illustrating the functional redundancy and adaptability of its repair systems (Brzostek et al., 2014).

Recent studies have revealed that *M. tuberculosis* encodes an even broader repertoire of DNA repair proteins than previously recognized. In addition to classical repair components, it possesses alternative enzymes such as Ligase C and the multifunctional polymerase Prim-PolC, which contribute to BER (Płociński et al., 2017). Furthermore, a non-canonical mismatch repair system involving NucS/EndoMS has been identified, which plays a compensatory role in the absence of classical MMR components. This pathway has been shown to participate in the correction of mismatches and prevent the accumulation of mutagenic lesions, further enhancing the bacterium capacity to adapt to genotoxic stress (Castañeda-García et al., 2017; Islam and Josephs, 2024).

In addition to protecting its own genome, *M. tuberculosis* has evolved strategies to interfere with host DNA repair mechanisms, thereby promoting its intracellular persistence. One such strategy involves the secretion of the UreC protein (Rv1850), which binds to the host protein RUVBL2, disrupting the formation of the RUVBL1-RUVBL2-RAD51 complex required for homologous recombination in host cells. This disruption impairs the host cell’s ability to repair its own DNA, leading to the accumulation of DNA fragments in the cytoplasm in the form of micronuclei. These micronuclei activate the cyclic GMP–AMP synthase (cGAS) and stimulator of interferon genes (STING) pathway, resulting in type I interferon (IFN-β) production and excessive formation of lipid droplets through the scavenger receptor A1 (SR-A1). These lipid droplets serve as nutrient reservoirs that facilitate bacterial growth within the macrophage (Liu et al., 2023).

Such dual functionality - both preserving bacterial DNA integrity and manipulating host cell responses - demonstrates how DNA repair systems serve as pivotal elements of bacterial survival and pathogenesis. While these mechanisms provide robust defense against genomic instability, they also present potential vulnerabilities. The molecular components of bacterial repair pathways are markedly different from their eukaryotic counterparts, making them attractive targets for novel antimicrobial therapies. Disrupting key repair enzymes in pathogens could sensitize them to host immune responses or enhance the efficacy of existing antibiotics. Moreover, the redundancy and versatility of these repair systems highlight evolutionary pressure on bacteria to adapt to rapidly changing and often hostile environments. These pressures are especially significant for bacteria residing in dynamic ecological niches - whether in soil, aquatic systems, or within hosts - where exposure to genotoxic compounds is common. Understanding these systems in detail not only reveals fundamental principles of microbial genome maintenance, but also informs clinical strategies for combating persistent infections.

## Metabolic signaling in bacteria: a central network for environmental sensing and adaptive control

As already mentioned, bacterial habitats are characterized by highly dynamic and fluctuating physicochemical conditions. A central component of the cell’s adaptive capacity is metabolism, defined as the entirety of biochemical reactions occurring within the cell, encompassing both; catabolic and anabolic processes.

Within the framework of central metabolism, carbon oxidation pathways - such as those involving glucose and organic acids - play a particularly prominent role. These pathways provide ATP and metabolic intermediates essential for downstream biosynthetic reactions. Importantly, metabolism serves not only as an energy provider but also as a central regulatory node that integrates environmental signals and coordinates cellular responses. This is achieved through modulation of gene expression, enzyme activity, and cellular architecture (Ledezma-Tejeida et al., 2021; Schink et al., 2022; Allen et al., 2023; Holbrook-Smith et al., 2024).

Bacterial capacity to finely tune metabolic fluxes in response to environmental cues exemplifies the sophistication of prokaryotic regulatory systems. Under nutrient-rich conditions, cells commit to growth and proliferation, whereas in adverse environments, they can transition into a metabolically quiescent state, conserving resources and enhancing stress tolerance. This metabolic flexibility is fundamental to bacterial survival and underpins the maintenance of internal homeostatic balance under fluctuating environmental conditions (Kleckner et al., 2018; Morcinek-Orłowska et al., 2019; Knöppel et al., 2023). An illustrative example of the integration of metabolism with the regulation of cellular responses to changing environmental conditions is the control of the intracellular level of the (p)ppGpp alarmone - a central regulator of the stringent response (Irving et al., 2021) (see above for details). It has been demonstrated for *E. coli* that the *ytfK* gene, which encodes a protein modulating SpoT activity, plays a pivotal role in the regulation of SpoT-dependent (p)ppGpp synthesis. The *ytfK* gene expression is strongly induced under glucose-limiting conditions and is dependent on the cAMP-CRP complex. Importantly, elevated levels of YtfK are sufficient to trigger SpoT-dependent (p)ppGpp accumulation even in the absence of external stress, indicating that YtfK functions as an intrinsic activator of this regulatory pathway (Meyer et al., 2021). Moreover, *ytfK* expression is responsive to a variety of environmental cues, including phosphate starvation (via the PhoR-PhoB system), iron limitation (via Fur), fatty acid starvation (through depletion of the acetyl-CoA pool), and oxidative stress (Iwadate and Kato, 2017; Meyer et al., 2021).

Mechanistically, YtfK binds to the catalytic domain of SpoT, while other effectors, such as the acyl carrier protein (ACP), interact with the regulatory TGS (ThrRS, GTPase and SpoT) domain. This suggests potential cooperative regulation of SpoT by distinct metabolic inputs. In addition, the cAMP-CRP complex, a canonical regulator of carbon availability, also contributes to translational control by integrating metabolic signals with the regulation of (p)ppGpp homeostasis. Under glucose-limited conditions, cAMP-CRP activates transcription of *relA* and *spoT*, and concurrently promotes translation of their gene products through induction of ribosomal protein S1 acetylation, a modification catalyzed by the CRP-dependent acetyltransferase YfiQ. This coupled regulatory system enables a rapid adjustment of gene expression in accordance with cellular energy status, thereby coordinating (p)ppGpp levels with overall metabolic activity. Notably, (p)ppGpp itself exerts negative feedback on CRP activity by directly competing with cAMP for CRP binding. This establishes a dynamic feedback loop that facilitates a rapid, switch-like, and population-wide adaptive response (Zhao et al., 2024). Collectively, this relationship demonstrates that metabolism not only reflects the energetic state of the cell but also actively shapes gene expression patterns and adaptive strategies by integrating transcriptional, translational, and metabolic signaling into a cohesive physiological response.

An important aspect of metabolic signaling in bacteria is the regulatory role of metabolites. Effective adaptation to constantly fluctuating environmental conditions requires that metabolite-mediated signals be transduced into dynamic changes in metabolic pathway activity. These pathways, composed of interconnected enzymatic reactions, are responsible for the synthesis and degradation of metabolites essential for sustaining core cellular processes. Their activity is regulated at two fundamental levels: (i) by modulating the transcription of genes encoding metabolic enzymes and (ii) through direct modification of enzymes’ catalytic properties, such as allosteric interactions or post-translational modifications (Radoš et al., 2022; Sporre et al., 2023; Gruber et al., 2025). In prokaryotic cells, central carbon metabolism intermediates - including pyruvate, α-ketoglutarate, and fumarate - have been shown to significantly mitigate the phenotypic effects of mutations in genes essential for DNA replication initiation and elongation (e.g., *dnaA, dnaB, dnaC, dnaG, dnaE*, and *dnaN*) (Krause et al., 2020). This finding suggests that these metabolites may serve as intermediate regulators that connect metabolic state to the replication machinery, modulating enzymatic activity or protein-protein interactions to ensure fidelity and coordination of DNA replication. Such mechanisms are crucial for maintaining genome integrity and DNA homeostasis under diverse physiological conditions.

Another well-characterized mechanism by which metabolites exert direct control over gene expression involves riboswitches - structured RNA elements typically located in the 5′ untranslated regions (5′ UTRs) of bacterial mRNAs. These RNA domains bind specific small molecules without the need for protein cofactors and subsequently modulate transcription or translation in response to intracellular metabolite levels (Nudler et al., 2004). A recently described Na^+^-sensing riboswitch, for example, regulates expression of genes involved in the sodium transport and homeostasis, highlighting the expanding diversity of ligands sensed by RNA-based regulators (White et al., 2022). Similarly, the characterized NA riboswitch family selectively binds purine nucleosides such as adenosine, 2′-deoxyadenosine and inosine to control genes involved in nucleoside transport and metabolism. Additionally, *ykkC* family riboswitches, which were previously associated with guanidine sensing, have been shown to include variants that recognize different nitrogen-containing compounds, further illustrating the ligand diversity within a single structural framework (White et al., 2022). Notably, some riboswitches have been shown to bind two distinct ligands within a single binding site, revealing increasingly complex modes of metabolite sensing by RNA (Ruff et al., 2016). For example, the *glmS* riboswitch ribozyme requires glucosamine-6-phosphate (GlcN6P) as a ligand and divalent metal ions (typically Mg^2+^) as essential cofactors for catalytic self-cleavage. Another example is the tetrahydrofolate (THF) riboswitch, which binds different folate derivatives in two separate ligand-binding sites, allowing fine-tuned control of folate-related gene expression (Ruff et al., 2016).

From a broader systems-level perspective, recent studies analyzing the condition-dependent metabolome of *E. coli* (Radoš et al., 2022) have also demonstrated that nucleotides and amino acids constitute the most stable metabolite classes across varying environmental conditions. This relative invariance supports the long-standing hypothesis that maintaining end-product homeostasis is a fundamental objective of biosynthetic pathway regulation (Hofmeyr and Cornish-Bowden, 1991; Schafer et al., 2004). Maintaining constant levels of major metabolites likely supports the efficient execution of fundamental processes such as nucleic acid and protein synthesis, despite fluctuations in metabolic flux.

Taken together, these examples - from riboswitch-mediated regulation to metabolite buffering of DNA replication - illustrate that metabolites act as rapid and versatile signaling molecules, enabling bacteria to tightly couple metabolic flux with gene expression and DNA replication (Radoš et al., 2022). Their direct interactions with transcription factors (Sauer and Eikmanns, 2005), riboswitches (Nudler et al., 2004; Ruff et al., 2016; White et al., 2022), and enzymatic effectors (Radoš et al., 2022; Sporre et al., 2023; Gruber et al., 2025) underscore their central role in maintaining cellular homeostasis and enabling precise adaptation to fluctuating environmental conditions.

## Iron homeostasis: hardwired to sense, collect, adapt and survive

Iron is a vital element for microorganisms due to its redox properties, which make it an essential cofactor for enzymes involved in central metabolic processes such as respiration, DNA replication and repair, and energy production (Frawley and Fang, 2014; Wofford et al., 2019; Zhang et al., 2020). Iron-dependent enzymes—including cytochromes, polymerases, and oxidoreductases—play indispensable roles in sustaining cellular function. However, due to its dual nature as both a critical nutrient and a catalyst of reactive oxygen species via Fenton chemistry and the Haber–Weiss reaction, intracellular iron concentration must be tightly regulated (Kwun and Lee, 2023). Excess iron promotes oxidative damage to DNA, proteins, and membranes, whereas iron limitation impairs the activity of key enzymes and restricts bacterial growth. Based on estimates, more than 6% of all genes in prokaryotes are transcriptionally responsive to iron levels (Smith et al., 2013), yet iron’s influence extends far beyond transcription. It modulates broader physiological traits including metabolism, quorum sensing, and virulence (Batista et al., 2024; Busch et al., 2025; Rios-Delgado et al., 2025; Weiner et al., 2025). To manage iron availability, bacteria have evolved complex regulatory networks that coordinate iron acquisition, storage, and utilization (Wofford et al., 2019; Baez et al., 2022). These systems function through integrated transcriptional and post-transcriptional mechanisms and are closely coupled to stress responses such as the stringent response (Vinella et al., 2005; Jordan et al., 2020; Zha et al., 2022).

A central component of iron-dependent gene regulation is the Ferric Uptake Regulator (Fur), a conserved transcription factor that dynamically responds to intracellular iron levels (Sevilla et al., 2021; Purcell et al., 2024). Under iron-replete conditions, Fur binds Fe^2+^ and forms a holo-Fur complex that represses genes involved in siderophore biosynthesis and iron transport by binding to Fur box elements in their promoters, preventing excess iron accumulation and oxidative stress (Sevilla et al., 2021). When iron is scarce, Fur dissociates from DNA, allowing derepression of iron acquisition genes. Beyond its classical role as a repressor, Fur can also act as a positive regulator—either directly by activating transcription, or indirectly by repressing small RNAs that would otherwise inhibit translation (Porcheron and Dozois, 2015; Purcell et al., 2024). For example, the well-characterized sRNA RyhB downregulates non-essential iron-using proteins and modulates iron uptake gene expression, helping to maintain intracellular iron homeostasis (Salvail et al., 2010; Tobe et al., 2014).

Given the importance of iron, many bacterial pathogens tightly link iron availability with virulence gene expression. Traits such as adhesion, invasion, and toxin production are activated in response to environmental cues, allowing bacteria to optimize their pathogenic potential during infection. One major host defense is nutritional immunity, a strategy by which the host restricts iron availability using proteins such as transferrin, lactoferrin, and lipocalin-2 (see review Healy et al. (2021)). As a result, pathogens must actively compete for limited iron resources, and their ability to sense, acquire, and utilize iron becomes a key determinant of virulence and survival within the host. Alongside canonical Fur orchestrated gene expression pathogenic strains utilize specific sRNAs to repress expendable iron-utilizing proteins, promotes siderophore production, and coordinates Fe-S cluster cofactor biogenesis (Kohli et al., 2025).

Recent findings in *Staphylococcus aureus* have demonstrated that iron limitation induces a sophisticated iron-sparing response mediated by the sRNA IsrR, which is tightly regulated by cellular iron status (Ganske et al., 2024). Under iron-depleted conditions, IsrR becomes derepressed and binds near the ribosome-binding sites of mRNAs encoding iron-containing proteins—especially TCA cycle enzymes such as aconitase (*citB*), succinate dehydrogenase (*sdhC*), citrate synthase, and transporter (*citZ*, *citM* respectively), *mqo*—and oxidative stress defense genes like catalase *katA*, leading to translational repression and, in some cases, mRNA degradation (Barrault et al., 2024a; Rios-Delgado et al., 2025). Interestingly, in contrast to other targets, IsrR binds the *katA* transcript upstream of the RBS (Rios-Delgado et al., 2025). This mechanism allows redistribution of iron toward essential functions, optimizing survival during host-imposed iron restriction. Whole-cell metal analyses suggest that IsrR enhances Fe uptake and increases intracellular pools of non-complexed iron. It also suppresses synthesis of the Fe–S cluster-containing methylthiotransferase MiaB, further conserving iron and improving fitness under iron-starved conditions (Barrault et al., 2024b). Moreover, proteomic data link IsrR activity to activation of the SaeRS virulence regulator, suggesting a dual role in metabolic adaptation and virulence control (Busch et al., 2025).

Another layer of this regulation is mediated by iron-responsive riboswitches, which function as *cis*-acting RNA elements capable of directly sensing iron ions and modulating gene expression at the post-transcriptional level (Kavita and Breaker, 2023). Traditionally associated with sensing cobalt and nickel, members of the NiCo riboswitch family have recently been shown to respond to Fe^2+^, revealing a broader metal-sensing specificity than previously assumed (Xu and Cotruvo, 2020). Structural and biochemical studies confirmed that certain NiCo riboswitches bind ferrous iron with physiologically relevant affinity, undergoing conformational rearrangements that repress or activate downstream genes (Xu and Cotruvo, 2022). Moreover, it has been shown recently that dual RNA regulation can occur via a *cis*-acting riboswitch and a *trans*-acting sRNA, forming an intricate regulatory network controlling essential metal transport genes (preprint (González-Espinoza et al., 2024). This finding reveals a riboswitch-based regulatory system that works independently of Fur and supports sRNA-driven control.

Finally, when faced with various environmental stresses, bacteria use iron-dependent sRNAs as a rapid adaptation mechanism that not only leads to altered metabolism, but also to transient antibiotic resistance (Xu and Lin, 2024; Ha et al., 2025). Under low iron conditions, RyhB downregulates respiratory metabolism and Fe–S cluster assembly genes, reducing the proton motive force and thereby limiting aminoglycoside uptake, such as gentamicin (Chareyre et al., 2019). Similarly, oxidative stress activates the OxyS sRNA, which upregulates the Fe–S cluster regulator IscR, shifting Fe–S biosynthesis from the ISC to the SUF pathway and increasing tolerance to aminoglycosides (Baussier et al., 2024). Additionally, redox-active metabolites like phenazines trigger metabolic alterations that enhance the activity of efflux pump and alters fluoroquinolone susceptibility (Gerstel et al., 2020). Even more importantly, resistance to the first-in-class siderophore cephalosporin cefiderocol (CFDC) has now emerged. In a clinical isolate of *A. baumannii*, whole-genome sequencing identified seven non-conservative missense mutations in iron transport-related genes—including *exbD*4, *tonB*2, *bauA*, *ftsI*, *piuA*, and *feoB*—associated with high-level CFDC resistance (MIC = 64 mg/L) (Strateva and Peykov, 2024). This marks one of the first reports of CFDC resistance linked to alterations in iron uptake pathways, highlighting the potential for treatment failure through disruption of the “Trojan horse” mechanism by which CFDC gains entry into bacterial cells. However, all these observations underscore a regulatory axis linking iron metabolism, sRNA-mediated signaling, and phenotypic antibiotic resistance, enhancing bacterial resilience under antimicrobial pressure.

## Toxin-antitoxin systems: intracellular time bombs in the service of the host bacteria

Toxin-antitoxin (TA) systems are genetic modules typically composed of two elements–a stable endogenous toxin and a labile antitoxin, which directly or indirectly neutralizes the toxin’s activity. Depending on the toxins’ mechanism of action, they have a potential to impair cell integrity or to disrupt crucial cellular processes, such as DNA replication or translation. This can result in either growth arrest or cell death. Consequently, precise and tight regulation of both elements is required to perceive homeostasis of the host cell. The expression of TA genes, as well as their interactions with other cellular components, are regulated by diverse molecular mechanisms at both the transcriptional and post-transcriptional levels (reviewed in (Jurėnas et al., 2022). A variety of environmental cues that influence the expression of bacterial toxin-antitoxin systems have been identified (recently reviewed in Ostyn et al. (2025)). This section outlines how attitude towards the role of TA systems has changed over time and presents a small selection of the latest well-documented examples illustrating how host bacteria can take over the biochemical functions of TA modules for different purposes, helping to maintain cell integrity and homeostasis, and enabling a quick response to changing environmental conditions.

**FIGURE 2:**
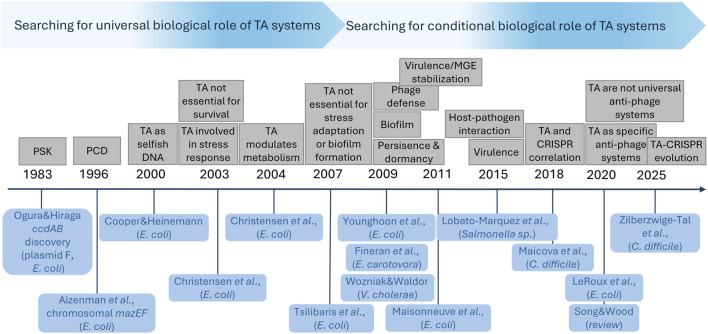
Illustration of gradual conceptual shift in perceiving of the biological function of TA systems. Various biological roles proposed for TA systems are listed above the timeline, while relevant articles mentioning for the first time a particular proposal are listed below the timeline. The spacing between tags on the timeline is not proportional to the time elapsed between individual publications.

The discovery of bacterial TA systems was made over four decades ago (Ogura and Hiraga, 1983). Because of their nature as self-regulated internal inhibitors of growth and their wide distribution in Bacteria and Archaea, they gradually became promising candidates for cell homeostasis modulators (Figure 2). Between 2000 and 2010 the predominant opinion in scientific community was that TA modules can be a crucial part of core biological processes in bacteria, affecting stress adaptation, persistence and dormancy (reviewed in: Gerdes et al. (2005), Van Melderen and Saavedra De Bast (2009), Yamaguchi and Inouye (2011), Cook et al. (2013), Park et al. (2013)). However, some data collected already in early 2000’s, indicated that chromosomally encoded TA modules of *E. coli* are not essential for cell survival during amino acid starvation, oxidative or temperature stress conditions nor for biofilm formation (Christensen et al., 2003; Tsilibaris et al., 2007). Eventually, after 2010 an increasing amount of contradictory data started to accumulate, especially from knock-out based studies, showing extremely high redundancy of TA modules (reviewed in: Fraikin et al. (2020), Song and Wood (2020), Jurėnas et al. (2022)). Moreover, it has been demonstrated, that TA systems are rather evolutionary unstable–they are frequently depleted or with limited (even strain-specific) occurrence (Habib et al., 2018; Fraikin et al., 2020). This has put the biological role of TA systems under an ongoing discourse. Nowadays, it is believed that TA operons, as a whole, are not universally essential (Song and Wood, 2020; Jurėnas et al., 2022), while antitoxin-encoding genes can be considered accessory essential genes (essential when the cognate toxin is present) (Rosconi et al., 2022). Nevertheless, it appears that particular TA systems, as conditional stress-response elements, can affect homeostasis of specific bacteria and be beneficial during growth under certain conditions. For instance, although it has been observed that multiple TA systems’ deletion in *E. coli*, *S*. *aureus, Salmonella enterica*, or *Pseudomonas putida* does not significantly affect bacterial fitness during growth under several stress conditions (Pontes and Groisman, 2019; LeRoux et al., 2020; Rosendahl et al., 2020), it has been also demonstrated that deletions of particular TA cassettes decrease survival of *Mycolicibacterium smegmatis* and *M. tuberculosis* under starvation conditions (Tiwari et al., 2015; Zhang et al., 2022). Likewise, deletion of multiple TA modules caused limited growth of *Enterococcus faecalis* during temperature and oxidative stress (Li et al., 2020). Interestingly, although, limited to specific cases, the influence of TA modules on bacterial cell homeostasis is incredibly versatile. It appears, that despite the fact that TA systems are not indispensable, they can relate to almost every aspect of bacterial biology affecting cell homeostasis, from modulation of mobile genetic elements stability and phage defence to second messengers’ activity and iron homeostasis maintenance.

Chromosomally encoded TA systems are predominantly located on genomic islands, such as prophages, integrative and conjugative elements (ICE), integrons, or transposons (reviewed in Jurėnas et al. (2022)). It has been demonstrated that TA modules promote stable maintenance of these genetic elements within the genome, ensuring genomic integrity and plasticity. For instance, antitoxins AbiEi and SezA of *Streptococcus suis* co-ordinately promote ICE stabilization and multidrug resistance by specifically binding to sequences in the *oriT* and *attL* sites, respectively (Gu et al., 2024). In addition, a number of TA systems of *Vibrio cholerae* modulates recombination dynamics of integron cassettes by an integrase-dependent mechanism termed “cassette loss killing” - the cell is killed by toxins when the overall cassette excision rate is too high. Since integrase expression is only observed under stressful conditions, bacteria generate diverse phenotypic combinations by reshuffling gene cassettes. This allows them to explore and select traits that best enhance their survival in specific environments (Richard et al., 2024).

Moreover, some TA systems have been linked to the DNA damage and repair processes. In diverse bacterial species, DarT toxins from the *darTG* TA systems function as ADP-ribosyltransferases that modify ssDNA in a sequence-specific manner, leading to DNA damage, SOS response, and inhibition of replication, while antitoxin DarG interacts with some DNA repair proteins, acting as a non-canonical repair enzyme (Zaveri et al., 2020; Weixler et al., 2021). Furthermore, in *M. tuberculosis*, *darTG* expression is co-regulated with the DnaB helicase, which is controlled by a DNA damage-inducible promoter. The DarT-mediated ssDNA modification at the origin of replication promotes slow growth and antibiotic tolerance of the host bacteria (Schuller et al., 2021). On the other hand, in uropathogenic *E. coli* (UPEC) the HipH toxin of an alternative TA-like system acts as a genotoxic deoxyribonuclease, inducing DNA double strand breaks and genome instability. In this module one of the second messengers, c-di-GMP, serves as an antitoxin exerting control over HipH expression and activity. This system is triggered by cell adhesion and regulates both bacterial persister formation in the presence of antibiotics associated with biofilms and the integrity of the bacterial genome (Liao et al., 2024).

Second messengers such as (p)ppGpp and (p)ppApp are also regarded as components of TA-like modules. However, in these cases it is the alarmone synthetases related to the Rel enzymes (RSH) that play a role of toxic components, as they produce molecules which are toxic to the cells. They are found to be genetically linked to the genes whose products act as antitoxins, although their mode of action requires further investigation. Given that many SASs and SAHs are encoded within prophage genomes or prophage-derived regions of bacterial chromosomes, they are thought to function either as superinfection inhibitors or as factors that evade the host defense systems (Jimmy et al., 2020; Hengge et al., 2023).

In recent years many new findings have emerged from studies of TAs as bacterial protection systems against phage infections (extensively reviewed in LeRoux and Laub (2022), Kelly et al. (2023)). It is now generally accepted that this might be a primary role of these chromosomally encoded entities, alongside with restriction-modification (RM) and CRISPR systems. Although there are many examples of different TA modules that inhibit phage propagation by inducing abortive infection (to save the bacterial population), only last year several excellent papers appeared describing actual molecular mechanisms underlying these processes. Two papers published in tandem in *Nature* show that upon phage infection (phage T7, T5 and T4), specific viral proteins (Ocr, Ptr and Arn, respectively) bind allosterically to the chromosomally encoded AriAB TA system protein complex (called PARIS) and cause a conformational change that releases the AriB toxin which then specifically cleaves tRNALys, thereby inhibiting translation and preventing production of new phage particles (Burman et al., 2024; Deep et al., 2024).

Furthermore, in some tripartite toxin-antitoxin-chaperone modules (TAC) the SecB-like molecular chaperone facilitates folding and protects the antitoxin from degradation and additionally acts as a viral infection sensor. For example, HigBAC expression is triggered by the gpV major tail protein of the lambda phage, which releases the HigB RNase toxin that restricts productive phage replication. Similarly, CmdTAC module detects viral capsid proteins to sense infection, while the CmdT ADP-ribosyltransferase toxin modifies mRNA to halt protein synthesis and limits 933W phage propagation (Mets et al., 2024; Vassallo et al., 2024). In addition, MqsRAC of *E. coli* was found to inhibit T2 phage development, but instead of leading to bacterial death to stop phage propagation, it was shown to induce persister cell formation (Fernández-García et al., 2024).

An interesting novel mode of anti-phage mechanism represents newly described ShosTA system that acts via DNA damage and repair. ShosT functions as both a phosphoribosyl transferase and a pyrophosphatase, disturbing purine metabolism by altering the nucleotide pool. This results in DNA duplications, cell filamentation, and ultimately cell death. In contrast, the ShosA antitoxin binds DNA and likely recruits additional factors to promote homologous recombination, thereby counteracting ShosT toxicity. Phage T7 protein Gp0.7, which is an inhibitor of the host RNA polymerase, was identified as a trigger for this TA module (Pu et al., 2025).

Overall, temperate phages are also a reservoir of anti-phage defense systems, including TA cassettes, that protect bacterial host against subsequent infection by unrelated temperate phages and/or mediate host defense against diverse lytic phages (Brenes and Laub, 2025). One such example is the tripartite KKP module, which consists of the Ser/Thr kinases PfkA and PfkB, which act jointly as a toxin, and the phosphatase PfpC, which serves as an antitoxin. These kinases have multiple host targets, including MvaU, a host nucleoid binding protein and prophage-silencing factor. This system also protects cells against certain lytic phages. The conserved phage replication protein Gp59 inhibits the PfpC phosphatase, thereby leading to activation of the toxic kinase and consequent suppression of phage replication. Thus, KKP functions as a phosphorylation-based strategy for both prophage control and antiphage defense (Guo et al., 2024). On the other hand, the chromosomal TA system MazEF has been co-opted by the temperate *Bacillus* phage ϕ3T to control the lysis-lysogeny decision via the arbitrium communication, which is a small molecule signalling system utilizing a hexapeptide (AimP). Upon infection, the MazF ribonuclease is activated by the combined action of several specific phage-encoded proteins. At low arbitrium signal concentrations, MazF is inactivated by two phage-encoded MazE homologues (AimX and YosL proteins), which allows the phage to complete lysis. AimX also inhibits the function of ϕ3T-93, a protein that promotes lysogeny by binding to MazE and releasing MazF. However, when the arbitrium signal is high, AimX is not expressed, so MazF remains active and lysogeny is promoted (Guler et al., 2024; Zamora-Caballero et al., 2024).

Interestingly, some HicA and RelB toxin genes were found by bioinformatic analyses in atypical genetic arrangements. For example, they are present in operons with prokaryotic Viperins (pVips) which produce modified nucleotides that block phage transcription, acting as chain terminators and therefore defend cells against bacteriophage invasion. However, how production and activity of these toxin RNases is regulated and if they contribute to the antiviral activity of pVips is unknown (Gerdes, 2024).

Finally, it has been demonstrated that in a number of bacterial species, specific TA modules play a regulatory role in the process of iron uptake during periods of stress. For instance, in an iron-limited environment the growth of *M. smegmatis* was strongly repressed upon *mazEF* expression. Furthermore, genes responsible for synthesis of iron-chelating siderophores, along with several genes involved in iron acquisition, transport, and storage, were significantly upregulated in the presence of MazEF. This demonstrates that *mazEF* expression mimics the iron-deficient conditions, resulting in activation of genes involved in iron uptake (Zhang et al., 2022). In a comparable fashion, activation of the MazF toxin in *Deinococcus radiodurans* in response to DNA damage downregulated Fur expression. This resulted in the derepression of Fur-regulated genes and enabled uptake of excess metal ions that triggered ROS-mediated cell death (Dai et al., 2021). Moreover, an increased expression of the *P. aeruginosa pacTA* TA genes has been observed in response to iron starvation. In this case, however, the PacT toxin, in addition to its typical function in arresting translation through acetylating aminoacyl-tRNAs, was capable of binding directly to the Fur protein, thereby inhibiting its DNA-binding affinity and unblocking expression of the genes involved in iron acquisition (Song et al., 2022). Furthermore, recent analyses of *Helicobacter pylori* have revealed that, under iron-rich conditions, Fur binds directly to the promoters of antitoxins of two putative type VIII TA systems, thereby repressing their transcription and liberating the toxic components of these systems (Vannini et al., 2024).

These findings show the remarkable plasticity of TA modules within the evolutionary context. It appears that while the TA systems probably exist in bacterial genomes mainly as selfish entities, they can be adapted to divergent functions depending on the specific habitat and requirements of their host, improving bacterial fitness. Interestingly, some TA systems can gain distinct functions in various bacteria. On the other hand, TA systems’ biological roles seem to be either dispensable or functionally overlapped by other genes, leaving open the possibility of TA encoding genes loss. It has been proposed that the decay of TA systems through loss of toxin’s toxicity results in their progressive loss during evolution (Fraikin et al., 2020). However, recent findings suggest that even inactive TA systems can provide selective advantages to the host. For example, the plasmid-encoded inactive TA module MtvTA in *P. aeruginosa* regulates plasmid conjugation and virulence. MtvTA represses plasmid transfer, enhances type III and IV secretion systems’ expression and promotes pyocyanin biosynthesis by directly activating specific promoters (Li et al., 2025). The evolutionary utility and universality of TA modules have been also demonstrated in a recent study, which shows that the Cas13 protein of RNA-targeting CRISPR-Cas system probably originated evolutionarily from the AbiF TA system (Zilberzwige-Tal et al., 2025).

To conclude, even though TA systems are not essential, they constitute a remarkably flexible genetic pool to form new components required for bacterial adaptation in the new ecological niches. Therefore, they are an additional important factor that, in certain cases, can significantly affect almost every aspect of bacterial cell homeostasis.

## Bacteriophages: maintaining homeostasis in the face of threats imposed by them on the cell

One of the factors with the potential to disrupt bacterial cellular homeostasis is the invasion by their natural pathogens - bacteriophages. Bacteriophages play a pivotal function in maintaining bacterial population homeostasis by regulating population size and nutrient availability. However, the infection of individual cells can result in cell death via the phage lytic cycle or may create a risk of destabilising the internal cell homeostasis. Consequently, bacteria have evolved diverse mechanisms to defend themselves against viruses. The majority of such mechanisms involves degradation of invading nucleic acids using either programmable, sequence-specific, or non-specific nucleases (Georjon et al., 2023).

An example of the defense mechanism, which has found application in molecular engineering, is the CRISPR-Cas system. Reverse transcriptases associated with CRISPR-Cas perform RNA-templated DNA synthesis to facilitate spacer acquisition directly from viral RNA transcripts (Silas et al., 2016). These transcripts are then used to degrade target phage DNA using other components of this system. Other defense-associated reverse transcriptase (DRT) systems encode and reverse transcribe bacterial non-coding RNAs (ncRNAs) (Mestre et al., 2020; Azam et al., 2024). In that case, retron-encoded ncRNAs serve as templates for the synthesis of hybrid RNA–DNA molecules—known as multicopy single-stranded DNA (msDNA) — that serve as antitoxins against host-encoded toxins, e.g., RT-msDNA antitoxin complex neutralizes the RcaT toxin in *Salmonella typhimurium*. Upon phage infection the RT-msDNA production is disrupted and the RcaT toxin is activated which leads to abortive infection and cell death (Bobonis et al., 2022). Another example is the *Klebsiella pneumoniae* DRT2 system that employs a reverse transcriptase which binds to a ncRNA. This system has been investigated in *E. coli*, where it was shown that upon T5 phage infection the ncRNA is reverse transcribed into an array of tandem repeats that reconstitute a functional *E. coli* promoter and open reading frame, allowing expression of a toxic Neo protein whose precise function remains to be elucidated, but which leads to abortive phage infection (Wilkinson et al., 2024). All the aforementioned DRT employing mechanisms lead to abortive infection via releasing host-encoded toxin from TA complexes, killing the infected cell and thus prevents further viral propagation what leads to homeostasis within the population albeit at the expense of the individual infected cell.

The invading phage DNA can be also captured by membrane-associated systems, such as the membrane-associated Kiwa system from *E. coli* which protects the cell against phage lambda and SECphi18 infections. This supercomplex consists of two factors: the KwaA transmembrane protein which becomes activated at the site of phage attachment, and the KwaB protein which facilitates subsequent binding of phage DNA. The phage replication and late transcription are then disrupted in cooperation with the bacterial RecBCD recombination system, without inducing cell death. In addition, even though both, the Kiwa and RecBCD systems are individually sensitive to the phage-encoded inhibitor such as Gam, their joint action creates a buffering effect, allowing one system to function when the other is inhibited (Zhang et al., 2025).

In the event of the antivirus defense systems’ failure and unfavorable environmental conditions for bacteria, some phage may remain latent within the cell, thus entering the lysogeny pathway (for the review see Howard-Varona et al. (2017)). In the lysogenic cycle the bacteriophage genome remains strictly integrated with the bacterial hosts’ genome. The survival of the phage in such a form is directly linked to the survival and condition of the host, thus resulting in a mutualistic interaction. In other words, bacteriophages appear to be interested in maintaining homeostasis within the host cell and ensuring that the host is interested in maintaining it.

Since many prophage genes contribute to their host’s physiology, they need to be expressed at appropriate time and the product they encode must reach appropriate location. This necessitates their incorporation into the bacterial regulatory network, thus ensuring maintenance of internal homeostasis. In order to achieve this, bacteria have developed systems of silencing such genes before being integrated into the host regulatory network. Silencing can be achieved through DNA modification (e.g., epigenetic silencing via modification of DNA by Dam methylation of phage promoters (Casadesús, 2016) or by regulatory proteins that bind DNA to prevent transcription, such as H-NS, MvaT and Lsr2 (Ali et al., 2012). This negative regulation concerns most of the phage genes, including those involved in the process of prophage excision (reviewed in Wahl et al. (2019)).

On the other hand, many bacteriophages encode genes which, although not directly involved in the phage life cycle, can enhance the bacterial host’s fitness. Such genes lead to lysogenic conversion and are termed “morons” (Cumby et al., 2012). The impact of morons is mainly concerned with bacterial virulence, metabolism, resistance to other phages or to phage super-infection, tolerance to various stresses, antibiotic resistance, and acquisition of new bacterial host features (for review see Tsao et al. (2018), Taylor et al. (2019)). These genetic traits can enhance bacterial survival and adaptability, thereby contributing to internal and external (population) homeostasis. For example, it is predicted for *Salmonella* spp. prophages that they provide such advantages to their host, as (i) modification of cell surface structures (i.e., glycosyltransferases); (ii) modulation of host responses (e.g., typhoid toxin); (iii) conferring resistance to heavy metals and antimicrobials; (iv) metabolism of carbohydrates, amino acids, and nucleotides; and (v) DNA replication and repair (Yates et al., 2024). This evidently indicates prophage influence on the metabolic, virulence, and resistance characteristics of *Salmonella*. New examples of the aforementioned functions are still being uncovered, but it is worth to elaborate on the classic example of glucosylation carried out by prophage-encoded proteins to modify the O-antigen in *Salmonella*. Glucosylation occurs according to phase variation, which is in turn regulated by the availability (via methylation) of the promoter for the prophage encoded operon responsible for this process. Such temporarily changing surface modification allows bacteria to escape the eukaryotic immune system and can prevent superinfection by phages that use similar O-antigen receptor (Davies et al., 2013; Wahl et al., 2019).

In addition, direct impact of phages (enhancement or inhibition) on three key forms of bacterial motility–twitching, swimming and swarming–has been confirmed. Prophages have been found to encode genes for bacterial adhesins, thereby increasing the virulence of the bacterial lysogens. Also, it is notable that phages encode some of the most dangerous virulence factors, known as toxins or effector proteins. These include toxins that cause severe pathologies, such as cholera, diphtheria and botulism. Furthermore, bacteriophages have been shown to play an active role in quorum sensing systems, which regulate a broad spectrum of genes involved in virulence, biofilm formation, motility, antibiotic resistance, metabolic pathways and lifestyle choices. Moreover, prophages have been demonstrated to encode genes that mimic host cell communication molecules (for review see Taylor et al. (2019), Zuppi et al. (2021)).

Finally, it is important to note that due to the high evolutionary capacities of phages and bacteria, phage–bacteria interactions may change very rapidly over time depending on environmental conditions. Recent research on the evolutionary dynamics of prophage maintenance in lysogen populations has revealed that prophage maintenance and loss is primarily determined by environmental conditions that alter the fitness benefits or costs of an active prophage. It has been observed that even in cases where prophages are costly and environmental selection pressure is against the prophage, if the bacteria receive prophage-encoded benefits, the prophage can nevertheless be maintained. Furthermore, prophages that encode genes exclusively benefiting the lysogen are maintained at higher frequencies than those benefiting the entire population (Bailey et al., 2024). In the event that the cost of maintaining the prophage becomes too high for the host, bacteria can mitigate those costs in several ways such as accumulation of deleterious mutations in the prophage, or mutations in the bacterial genome that alter phage induction rate. Bacteria can also eliminate prophages from their genomes by way of incomplete activation or complete deletion (Bailey et al., 2024).

## Biofilms: an example of multicellular prokaryotic homeostasis

Bacteria, while studied in the laboratory environment and cultivated in liquid media, have been viewed for a long time as free-living planktonic unicellular organisms. However, subsequent studies revealed that it is the sedentary and organized in multicellular communities lifestyle that is the most common for bacteria. Moreover, research initiated by Bill Costerton in the 1970s, who was a pioneer in biofilm studies, resulted in the disquieting conclusion that over 80% of bacterial diseases are associated with biofilm (Costerton, 1999; Flemming and Wuertz, 2019). The transition from planktonic to biofilm lifestyle requires major alterations in metabolism and energy-related processes, accompanied by structural modifications which require an intricate and well refined shift of balance in regulatory networks. Above all, this shift is generally dictated by the nutrient availability and subsequent onset of stress in order to balance growth and maintenance in a resource-limiting environment, as elegantly presented in the review by (Hengge, 2020). The intense proliferation of bacterial cells under nutrient-abundant conditions (e.g., exponential growth in the defined media) is a highly unstable situation and any fluctuations to suboptimal and further unfavorable conditions lead to a cascade of effects at the gene expression level, starting with the onset of stationary phase.

This transcriptional switch facilitates stress resilience which is directly and indirectly mediated by global alterations in gene expression that in the *E. coli* model bacterium are exerted by a specific sigma factor, σ^S^ (Hengge, 2014; Browning and Busby, 2016). Here, key roles in redirecting transcription machinery to σ^S^ dependent regulons are played by second messengers. For example, (p)ppGpp affects competition between σ subunits for core RNA polymerase such that alternative σs (including σ^S^) can outcompete the house-keeping σ^D^ factor and direct the RNA polymerase holoenzyme to specific promoters. This also leads to activation of the *rpoS* gene expression (encoding σ^S^). In addition, (p)ppGpp facilitates expression of DsrA, a small regulatory RNA necessary for efficient *rpoS* mRNA translation (Girard et al., 2018; Hengge, 2020). Moreover, the balance between (p)ppGpp and cAMP reflects homeostasis between stress and nutrient limitation induced shutdown of energy consuming processes such as growth (promoted by (p)ppGpp), and utilization of a variety of energy sources necessary for growth under non-optimal conditions (mediated by cAMP). In addition, the regulatory cascade, driven initially by σ^S^-mediated transcription, activates the expression of another transcription factor, CsgD, which is a key player in inducing the synthesis of extracellular matrix and promoting multicellular structures (Brombacher et al., 2003; Ogasawara et al., 2011). Another important second messenger in biofilm formation is c-di-GMP, regulating the cellular lifestyle switch by complex signaling pathways. One of the c-di-GMP-dependent effects involves changes in flagella rotations, diminishing cell motility and facilitating bacterial attachment to the surface (Boehm et al., 2010; Serra and Hengge, 2019).

The important part of bacterial cell-to cell communication is the quorum sensing (QS). This system is based on specific signals, such as the acyl homoserine lactone in Gram(−) bacteria and the specific peptide system in Gram(+) bacteria (Preda and Săndulescu, 2019). Primarily, QS is important to trigger the events leading to the switch from free-living to sedentary status, communicating the population density (Parsek and Greenberg, 2005; Zhou et al., 2020). In addition, these systems are involved in modulating gene expression, chemotaxis, virulence, signal transduction, acquisition of nutrients and secretion of secondary metabolites (for review see Waters and Bassler (2005), Mukherjee and Bassler (2019)). Thus, in the mature biofilm QS plays also a communication role to establish relevant connection between bacteria by exchanging signal molecules, and controlling the sharing so called “public goods” between producers and non-producers (Drescher et al., 2014; Mukherjee and Bassler, 2019). Also, limited biofilm disassembly to disperse microorganisms to other locations is mediated also by QS (Solano et al., 2014).

The ultimate goal of the life-style transition in bacteria is to establish a low-profile strategy which constitutes a trade-off between growth and survival (Ferenci, 2016). The most important factors in this strategy are protection from adverse conditions and establishing homeostasis not only within a single cell, but within biofilm as a whole. Such protection is secured by extracellular matrix which consists of exopolysaccharides, proteinaceous material, extracellular DNA (eDNA), fibers called curli and modified phosphoethanolamine (pEtN) cellulose (Barnhart and Chapman, 2006; Flemming and Wuertz, 2019; Buzzo et al., 2021). Bacteria encased in this matrix comprise a dynamic system, allowing to exchange of genetic material and molecular signals. Moreover, cells at different locations inside biofilm may have distinct metabolisms (e.g., different transcriptomic and proteomic profiles) due to intrinsic chemical gradients (Stewart and Franklin, 2008). As the biofilm microcolony grows and matures, bacteria within the matrix respond to signals from their surrounding environment, which eventually leads to dispersion of a portion of the encased bacteria (Kaplan, 2010; Kostakioti et al., 2013). Dispersed bacteria can return to planktonic form or continue the process of biofilm formation on new surfaces. Thus, the intra-biofilm homeostasis allows bacteria to maintain the biofilm specific structure while still keeping the ability to colonize other locations.

Although biofilms evolved to diminish bacterial vulnerability to environmental cues, these multicellular structures provide not only physical protection but also play other functions. For example, recent studies indicate that biofilm-embedded bacteria actively and dynamically affect their environment by resource management, maintaining redox balance, creating chemical gradients and counteracting the adverse effects of external conditions. They thus construct their own environment which provides better chances of their survival. In fact, the niche construction theory, originally proposed for eukaryotic organisms (Turner, 2016), has been extended to multicellular bacterial communities, as it follows similar principles and thermodynamic rules (Hengge, 2020).

The complex and functional biofilm organization provides physical, metabolic and functional resistance to various compounds, including antibiotics, disinfectants, or their host’s immune system defense. These features allow bacteria to survive under diverse conditions, including deep sea with anaerobic and high-pressure environment (Hadfield et al., 2025), industrial systems, soil, sediments and many others (Sentenac et al., 2022). Most importantly, biofilm-associated infections and contamination of medical devices and surfaces comprise a serious threat in current medicine and result in treatment difficulties of biofilm-related chronic infections (Høiby et al., 2011). Therefore, understanding factors responsible for homeostasis within the biofilm is crucial for designing effective antibiofilm strategies. Furthermore, in the case of biofilms associated with living organisms, there is an additional level of interaction, i.e., interaction between the biofilm and the host. The status of a given biofilm–whether beneficial or harmful–usually is not arbitrary and depends on the delicate balance between the status of both sides (e.g., biofilms in the oral cavity) (Zhao et al., 2023). At this level, homeostasis is achieved by exchanging signals between biofilm and the host, a state that can easily change in e.g., dysbiosis. Amongst biofilm-related infections, numerous are well-described, e.g., *P. aeruginosa* infections in cystic fibrosis or the urinary tract infections caused by UPEC strains (Vestby et al., 2020). Interestingly, a recent report indicated there are specific biofilm-associated molecular patterns (BAMPs) expressed in biofilm during infections that affect the host’s innate immune response (Østrup Jensen et al., 2025). This shows that the interactions between biofilm and its host can be even more complex and will require future work to be fully understood.

Yet another level of complexity is exemplified by multispecies biofilms. In fact, such biofilms are quite common and involve not only various bacterial strains, but also archaea, fungi and viruses. The co-existence in such structures requires communication between bacteria with different requirements and gene expression patterns, however once homeostasis is established, bacteria can benefit from protection ensured by the matrix, increased antibiotic resistance or higher sensitivity to outer signals (Anju et al., 2022; Kulshrestha and Gupta, 2022). Also, the heterogenicity in biofilms are proposed to facilitate diversity and bet-hedging strategies (Morawska et al., 2022). The cells, embedded in a relatively safe environment of biofilm, can sustain the effects of spontaneous mutations, which might result in their suboptimal response to current conditions. However, such mutation, if maintained, may provide selective advantage for possible future challenges. Thus, biofilms may be considered as a crucial part in the strategy to ensure evolutionary benefits for the entire population.

## Concluding remarks

**FIGURE 3:**
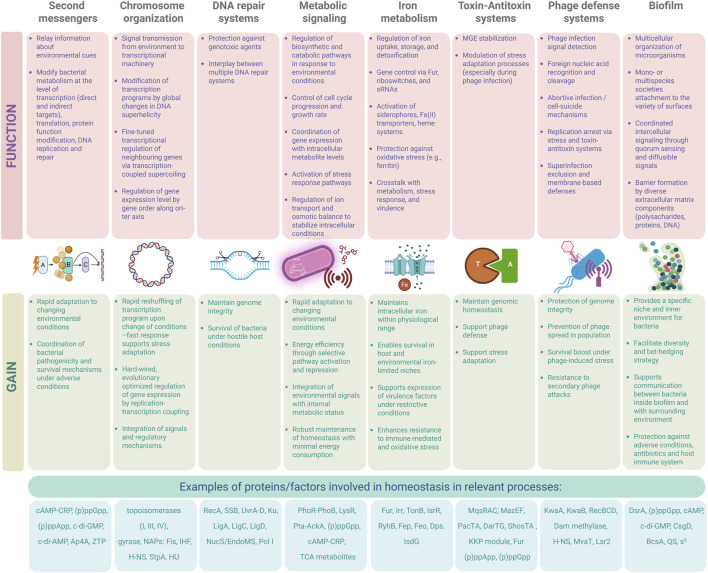
Overview of bacterial homeostasis systems: functions and adaptive advantages. The scheme summarizes the major prokaryotic systems involved in maintaining cellular homeostasis, organized by functional category. Each module is described by its core biological functions (top row) and the adaptive advantages it confers under environmental and host-related stress conditions (middle row). In the bottom row, major factors playing crucial roles in above-described processes are listed. Together, these systems orchestrate bacterial survival, stress responses, and pathogenesis across diverse niches. Created in BioRender. Szalewska-Pałasz (2025) https://BioRender.com/dbx16my.

Bacteria have evolved intricate regulatory networks to preserve their cellular homeostasis, ranging from sensitive systems to detect environmental conditions such as nutrient availability, to those allowing them to evade DNA damage, bacteriophage infection, and antibacterial agents (summarized in Figure 3). Preserving homeostasis is the most important basis of their life-style flexibility, thus understanding these processes is indispensable for both the basic and applied sciences. These studies may be accelerated by increasingly new ways and broad approaches to study basic cellular processes, e.g., DNA replication or iron homeostasis (reviewed in Maciąg-Dorszyńska et al. (2025) and Strzelecki and Nowicki (2025), respectively). Other aspects, such as understanding how chromosomal architecture and DNA topology coordinate global gene expression are essential for optimizing strain engineering and synthetic biology applications (Hustmyer and Landick, 2024), as is understanding of how this architecture is governed under changing environmental conditions (reviewed in Glinkowska et al. (2021)). Also, regulation of multiple processes to ensure optimal growth and adaptive responses requires complex mechanisms integrated into gene regulatory networks (GNRs), allowing efficient flow of information and relevant reactions at the molecular levels. These networks provide optimized energy consumption vs. maintenance balance; the concept of GNRs and their further implications in cellular homeostasis has been already introduced with mathematical models presented (Nagy-Staron et al., 2021; Graafland and Gutiérrez, 2022; Gupta et al., 2022); yet, additional research is necessary for thorough understanding of these processes.

As the cellular homeostasis has been reported to be a basis for antibiotic resistance for important pathogens (Rivera-Galindo et al., 2024; Salzer et al., 2025), targeting homeostasis regulatory networks to combat pathogens is an attractive possibility in the era of increasing bacterial antibiotic resistance, e.g., (p)ppGpp metabolism and the stringent response has been a focus of such studies in the past several years, as is the use of the toxin-antitoxin systems, bacteriophages and iron-uptake systems to design prospective therapies (reviewed in Gąsior et al. (2025), Boss and Kędzierska (2023), Karczewska et al. (2023) and Strzelecki et al. (2025), respectively).

Certainly, bacteria are perfect models not only to study distinct processes, as it has been generally assumed, but also to reveal complex mechanisms leading to the homeostasis at the level of single cells in challenging environments, as well as in the interactions with other cells and higher organisms. Further studies on the molecular mechanisms of homeostatic regulation are expected to provide information on the novel targets for efficient antimicrobial therapies.
